# Editorial

**Published:** 1868-06-01

**Authors:** 


### *EDITORIAL.*

We surrender a considerable proportion of the present number of the Journal to the triennial catalogue and announcement of Rush Medical College, as containing matter of permanent interest to the profession, and in order to reach as large a number of readers as possible, Twelve thousand copies are to be issued, and, as the forms are stereotyped, as many more as we can find names to address. The attention of all graduates of Rush Medical College is called to the published list of the *alumni*, and if any mistakes are noticed, a favor will be conferred in pointing them out. Prior to the reorganization of the College, the books were kept with a disregard of order, and of a high standard of Medical Education somewhat wonderful to contemplate. Hereafter it is believed that errors will be few or none.

Old subscribers to this Journal, will not need to be informed that the present editor will not deprive them of their due amount of reading matter by crowding upon it this time. The space will be returned them soon.

Items, News and Gossip. The Regents of the University of Michigan have applied to the Supreme Court for a *mandamus* to compel the State Treasurer to pay over the *pottage* for which they recently sold out the birthright of the Medical Department. Several of our exchanges are severe on Profs. Palmer, Douglass and Sager for the vulgar tenacity with which they adhere to the floating fragments of the former College at Ann Arbor. We sincerely trust that our *confreres* will forbear until it is ascertained why the American Medical Association happened to admit delegates from the present hybrid concern to their late meeting.—*Nous Verrons.*——The *Richmond Journal* has been removed to Louisville, Ky., the editor, Dr. Gaillard, having been appointed to a professorial chair in that city. ——The State Medical Society of Ill., at its recent meeting, failed to pass the resolutions introduced by Dr. Haller last year, taking possession of the medical colleges in the West. It is understood the Apostle refuses to be comforted. Details of its doings next number.——Prof. Freer has returned to Europe for the summer. He will keep our readers duly posted on medical matters over the water.——Prof. Gunn has removed his office to the Medical College.——The homœopathists of the North-west censure their Michigan brethren for accepting an organization of their craft away from Ann Arbor.——It is understood that the proprietors of the recently seized vinegar factory in this city, and the Cleveland Homœopathic School have tendered the Regents of the University of Michigan a consolidation with the Ann Arbor concern. Hybrids are not prolific.

